# Pooled analysis of the association between food insecurity and violence against women: Evidence from low- and middle-income settings

**DOI:** 10.7189/jogh.13.04021

**Published:** 2023-03-10

**Authors:** Rachel Jewkes, Esnat Chirwa, Deda Ogum Alangea, Adolphina Addo-Lartey, Nicola Christofides, Kristin Dunkle, Leane Ramsoomar, Andrew Gibbs

**Affiliations:** 1 Gender and Health Research Unit, South African Medical Research Council, Pretoria, Gauteng, South Africa; 2 School of Public Health, Faculty of Health Sciences, University of the Witwatersrand, Johannesburg, Gauteng, South Africa; 3 Office of the Executive Scientist, South African Medical Research Council, Pretoria, Gauteng, South Africa; 4 School of Public Health, University of Ghana, Accra, Ghana; 5 School of Public Health and Health Systems, University of the Pretoria, Pretoria, Gauteng, South Africa; 6 Department of Psychology, University of Exeter, Exeter, Devon, UK

## Abstract

**Background:**

Intimate partner violence impacts relationships across the socioeconomic spectrum, nonetheless its prevalence is reported to be highest in areas that are most socio-economically deprived. Poverty has direct and indirect impacts on intimate partner violence (IPV) risk, however, one of the postulated pathways is through food insecurity. The aim of this paper is to describe the association between food insecurity (household hunger) and women’s experiences, and men’s perpetration, of intimate partner violence and non-partner sexual violence in data from Africa and Asia.

**Methods:**

We conducted a pooled analysis of data from baseline interviews with men and women participating in six Violence Against Women prevention intervention evaluations and present a meta-analysis using mixed-effects Poisson regression models. Data were from South Africa (two studies), Ghana, Rwanda (two data sets), and Afghanistan and comprised interviews with 6545 adult women and 8104 adult men. We assessed food insecurity with the Household Hunger Scale.

**Results:**

Overall, 27.9% of women experienced moderate food insecurity (range from 11.1% to 44.4%), while 28.8% of women reported severe food insecurity (range from 7.1 to 54.7%). Overall food insecurity was associated with an increased likelihood of women experiencing physical intimate partner violence, adjusted incidence rate ratio (aIRR) = 1.40 (95% CI = 1.23 to 1.60) for moderate food insecurity and aIRR = 1.73 (95% CI = 1.41 to 2.12) for severe food insecurity. It was also associated with an increased likelihood of men reporting perpetration of physical IPV, with aIRR = 1.24 (95% CI = 1.11 to 1.39) for moderate food insecurity and aIRR = 1.18 (95% CI = 1.02 to 1.37) for severe food insecurity. Food insecurity was not significantly associated with women’s experience of non-partner sexual violence, aIRR = 1.27 (95% CI = 0.93 to 1.74) for moderate or severe food insecurity vs none, nor men’s perpetration of non-partner sexual violence aIRR = 1.02 (95% CI = 0.90 to 1.15).

**Conclusions:**

Food insecurity is associated with increased physical intimate partner violence perpetration and experience reported by men and women. It was not associated with non-partner sexual violence perpetration, although there was some evidence to suggest an elevated risk of non-partner sexual violence among food-insecure women. Prevention programming needs to embrace food insecurity as a driver of intimate partner violence perpetration, however, non-partner sexual violence prevention needs to be shaped around a separate understanding of its drivers.

Intimate partner violence (IPV) prevalence is higher among women living in poverty in low- and high-income settings and poverty is now a well-recognised driver of IPV, despite the agreement that IPV can affect women of all levels of social status [1-3]. Evidence suggests that poverty has both direct and indirect impacts on IPV risk. Poverty, particularly acute poverty manifested as food insecurity, often causes conflict within a relationship over access to and use of resources, and it may enhance gender-norm-related stress following from the male partner’s inability to fulfil a provider role and feel affirmed as a man, as well as general stress, including over finances, with a greater propensity for conflict to escalate to violence [4-7]. The chronic stress of poverty, as well as experience of IPV and other traumatic experiences, increases the likelihood of depression and anxiety, which may both increase the likelihood of further IPV [8]. There may be increased arguments over sex and forced sex, due to the impact of stress on libido and jealousy over women earning money or suspicion about the possibility that their source of money might be transactional sex [4]. Harmful alcohol use and drug use are also commonly associated with poverty and stress and themselves increase the risk of IPV [9]. Poverty may also prolong women’s time spent in violent relationships due to reliance on resources received from a partner, holding more conservative views on gender and relationships, and may reduce women’s options for leaving the relationship [6].

Poverty also has multiple indirect impacts and thus present food insecurity may be an indicator of much more long-standing difficulties in accessing resources [1,10]. Childhood poverty is associated with much poorer educational outcomes, and these in turn impact future earnings [10]. Poverty in childhood is also associated with the social learning of aggression and emotional dysregulation, which impacts interpersonal conflict management and often results in poor communication skills and practices [11]. Women and men raised in poverty both tend to have more conservative views on gender relations and permissive views on the use of violence, which may reduce women’s ability to leave violent relationships or reject violent partners (or potential partners) [12-15]. Further, poverty is associated with a greater risk of exposure to child abuse and neglect, with consequent poor mental health, and substance abuse [16-19].

Although poverty has been recognized as both a driver and outcome of violence against women and girls (VAWG), the majority of studies to date have focused primarily on IPV and have used multiple measures of exposure and outcomes, hindering comparison across settings. Food insecurity, often referred to and assessed as household hunger (defined as having insufficient food to eat at a household level), is a manifestation of extreme poverty and acutely impacts those affected [20]. There is a growing body of research that has examined the association between food insecurity and experience and perpetration of IPV and non-partner sexual violence (NPSV), much of which was recently summarized by Hatcher et al. [6]. It has been complemented by important insights from qualitative research [4]. To strengthen the evidence base from low- and middle-income countries (LMICs), and advance the current literature base on the role of food insecurity in both IPV and NPSV experience and perpetration, we undertook a pooled analysis of baseline data using comparable measurement methods from six IPV prevention studies conducted in three countries in Africa (South Africa and Rwanda, both having two data sources, and Ghana) and one conflict-afflicted country in Central Asia, Afghanistan. The study’s IPV outcome used in this paper, as available, was the past 12 months of physical IPV and NPSV experience for women and perpetration for men. The paper aims to answer the following research questions: (i) does experience of food insecurity increase women’s risk of experiencing IPV and NPSV?; (ii) is food insecurity associated with men’s perpetration of IPV and NPSV?

## METHODS

The studies included in this pooled analysis were conducted under the UK-Aid funded What Works to Prevent Violence Against Women and Girls? Global Programme (What Works). The primary goal of What Works was to advance the evidence base on the prevalence and drivers of VAWG, and the effectiveness and costs of interventions to prevent VAWG. The current study uses the baseline data from 8104 men and 6545 women from six VAWG prevention studies in four countries (South Africa, Ghana, Rwanda, and Afghanistan) to assess the association between food insecurity and IPV and NPSV perpetration among men and experience among women (**Table 1**). These studies were the evaluations of the Stepping Stones and Creating Futures intervention (SSCF) (South Africa) [21], the Sonke Change intervention (South Africa) [22], Rural Response System (RRS) community intervention (Ghana) [23], Indashyikirwa couples intervention (Rwanda) [24] with accompanying evaluation for Indashyikirwa community-level impact (Rwanda) [25] and the Women For Women International (WFWI) Intervention (Afghanistan) [26]. The two South African studies were located in areas with very limited infrastructure, considerable informal housing and high levels of material deprivation. The studies in Rwanda, Ghana and Afghanistan were conducted in rural communities and small towns (Ghana). Women in the Afghan study were known to be more resource-poor when invited to participate.

**Table 1:** Data sets used for men and women included in the pooled analysis

	Study	Country	Study design	Number of clusters	n (men)	n (women)	Sampling or recruitment strategy	Age (years)
1	Evaluation of Stepping Stones and Creating Futures	South Africa	RCT	34	674	677	Study volunteers	18-35
2	Evaluation of Sonke Change	South Africa	RCT	18	2406	-	Study volunteers	18-45
3	Evaluation of the Rural Response System	Ghana	Quasi-experimental study	40	1973	1877	Household- based random sample survey	18+ (men) 18-45 (women)
4	Evaluation of Indashyikirwa couples	Rwanda	RCT	28	1651	1660	Volunteer recruitment from savings and loan association groups	18-50
5	Evaluation of Indashyikirwa community intervention	Rwanda	RCT	28	1400	1400	Household-based random sample survey	18-49
6	Evaluation of the social and economic empowerment intervention of Women For Women International	Afghanistan	RCT	6	-	1461	Study volunteers	18-49

RCT – randomised control trial

### Measures

We assessed socio-demographic characteristics for men and women, including, age, current marital status, and education. All studies except Indashyikirwa in Rwanda asked whether participants had worked in the past three months. Food insecurity was assessed using the three questions of the Household Hunger Scale [20]: in the past four weeks, how often was there no food to eat of any kind in your house because of a lack of money?; how often did you or any member of your household go to sleep hungry because of a lack of food?; how often did you or any of your household go a whole day and night without eating because of lack of food? The latter question was asked in all studies except Rwanda. The items were recoded as none / little, moderate and severe. This is an easy-to-use, well-validated measure [20]. Three level and binary measures of food insecurity were derived from the mean value of the three food insecurity items. As recommended by the scale developers, the following cut-offs were used for the 3-level food insecurity measure: 0 to 0.7 = no / little food insecurity; > = 0.7 to 1.7 = moderate food insecurity; > = 1.8 to 3.0 = severe food insecurity [20]. For the binary exposure, we combined moderate and severe food insecurity. The measures of IPV and NPSV are described in **Table 2**. We did not ask about NPSV in Rwanda because it was not a target of the intervention, nor in Afghanistan because of concerns about the particular sensitivity of the questions in that context.

**Table 2:** Violence against women measures

Physical IPV perpetration	Five items asking in the last 12 months. How many times did you: slap or throw something at her which could hurt (your wife or current girlfriend)?; push or shove?; hit with a fist or with something else which could hurt her?; kick, drag, beat, choke or burn?; threaten to use or actually use a gun, knife or other weapon?. Responses: “never”, “once” “a few times” or “many times”. Coding never vs. “once” or more to an item. The items were developed during the WHO Women’s Health and Domestic Violence survey, modified to assess men’s perpetration [27,28].
Physical IPV experience	As for perpetration, but questions asked about women’s experiences from a current or previous husband or boyfriend.
NPSV perpetration	Five items asking about NPSV perpetration in the last 12 months. How many times have you: forced or persuaded a woman or girl who was not your girlfriend or wife at the time to have sex with you?; tried to force or persuade any woman or girl who was not your girlfriend or partner to have sex with you, but did not succeed?; had sex with a woman or girl who was not your girlfriend or wife when she was too drunk or drugged to stop you?; have you and other men ever had sex with a woman or girl at the same time who was not your girlfriend or wife when she did not agree to sex or you forced her? Or done this when she was too drunk or drugged to stop you? Coded never vs. one or more times. The scale was first developed in South Africa and subsequently refined and used extensively in Asia-Pacific region[29].
NPSV experience	Women were asked about their experience in the past 12 months in six questions. How many times has any man who is NOT your boyfriend or husband forced or persuaded you to have sex against your will?; tried to force you to have sex against your will and did not succeed?; forced you to have sex against your will when you were too drunk or drugged to refuse?; did two or more men force you to have sex with them at the same time against your will?; did two or more men force you to have sex with them at the same time against your will when you were too drunk or drugged to refuse?; was there an occasion when you agreed to have sex with one man and one or more others who you had not agreed to have sex with forced you to have sex with them as well? This was coded in the same way as for the men.


IPV – intimate partner violence, NPSV – non-partner sexual violence

### Data analysis

Descriptive statistics were used to summarize participants’ socio-demographic characteristics within each study and in the pooled data. Standard errors for specific studies and pooled estimates took account of any stratification or clustering due to each study’s sampling procedures and pooled estimates were weighted according to the study sample size. Forest plots, I-square and Cochran’s Q statistics were used to assess the consistency of outcomes across the studies and the I-square values showed high heterogeneity in physical IPV experience/perpetration as an outcome (80%, *P* < 0.001 for men; 62.9%, *P* = 0.029 for women). Generalized Linear Mixed Effects Models were then used to estimate overall effects and account for any heterogeneity across the studies due to methodological diversity. One-stage Individual Patient Data (IPD) meta-analysis was performed using mixed-effects Poisson regression models was used to account for within-and between-study variances (heterogeneity) across studies for both men and women [30]. Study-specific estimates were derived from a post-estimation model of the mixed-effects Poisson regression model. Both the main and post-estimation models included participants’ age and childhood trauma experience as fixed effects. To assess the robustness and consistency of the results, we repeated the analysis using mixed-effects logistic regression models. All data were analyzed using Stata Statistical Software: Release 17. College Station, TX: StataCorp LLC. StataCorp. 2019 and all tests were interpreted at a 5% significance level.

## RESULTS

### Socio-demographic characteristics

Across the samples of the five studies with women, 70.4% were 18-35 years old. Over half (60.4%) of women were married to men, 35.5% were in a relationship, and just 4.1% were not in a relationship (**Table 2**). Only a third (33.7%) of the women had completed secondary school education or above, and a quarter (26.8%) had no schooling. Most women (55.0%) reported working in the past three months, though this was not asked in Rwanda because the targeted communities were primarily engaged in subsistence agriculture. A third of women (33.2%) reported experiencing past-year intimate partner violence, with study-specific prevalence ranging from 15.5% (Ghana) to 59.5% (SSCF). Across the two studies where NPSV was reported (SSCF and RRS Ghana), 11.0% of women reported experiencing past year NPSV, ranging from 2.9% in Ghana to 33.7% in SSCF. Overall, 27.9% of women reported moderate food insecurity (range from 11.1% to 44.4%) and 28.8% of women reported severe food insecurity (range from 7.1% to 54.7%) (**Table 3**).

**Table 3:** Socio-demographic characteristics of study participants

	Stepping Stones, South Africa	RRS, Ghana	Indashyikirwa couples, Rwanda	Indashyikirwa-community, Rwanda	WFWI, Afghanistan	Sonke Change, South Africa	All studies
**Women**	(n = 677)	(n = 1877)	(n = 1660)	(n = 1399)	(n = 932)		(n = 6545)
Age group							
*18-25 years*	455 (67.2)	561 (29.9)	265 (16.0)	217 (15.5)	218 (23.4)		1716 (26.2)
*26-35 years*	222 (32.8)	727 (38.7)	852 (51.3)	697 (49.8)	395 (42.4)		2893 (44.2)
*36-45 years*	0 (0.0)	458 (24.4)	488 (29.4)	401 (28.7)	318 (34.1)		1665 (25.4)
*46-49 years*	0 (0.0)	131 (7.0)	55 (3.3)	84 (6.0)	1 (0.1)		271 (4.2)
Current marital status							
*Married*	29 (4.3)	1068 (56.9)	1096 (66.0)	828 (59.2)	932 (100)		3954 (60.4)
*In a relationship*	524 (77.4)	664 (35.4)	564 (34.0)*	570 (40.8)*	0 (0.0)		2322 (35.5)
*No relationship*	124 (18.3)	145 (7.7)	0 (0.0)	0 (0.0)	0 (0.0)		269 (4.1)
Education							
*None*	0 (0.0)	401 (21.4)	288 (17.4)	240 (17.2)	822 (88.5)		1751 (26.8)
*Primary school*	56 (8.3)	426 (22.7)	1115 (67.2)	911 (65.2)	75 (8.1)		2583 (39.5)
*Secondary school or above*	621 (91.7)	1050 (55.9)	257 (15.5)	247 (17.7)	32 (3.4)		2207 (33.7)
Food insecurity							
*None/little*	144 (21.3)	1536 (81.8)	327 (19.7)	259 (18.5)	567 (60.8)		2833 (43.3)
*Moderate*	163 (24.1)	208 (11.1)	702 (42.3)	621 (44.4)	134 (14.4)		1828 (27.9)
*Severe*	370 (54.6)	133 (7.1)	631 (38)	519 (37.1)	231 (24.8)		1884 (28.8)
Employed in the past three months	173 (25.6)	1174 (62.7)	n/m	n/m	567 (60.9)		1914 (55.0)
Employed in the past year	97 (14.3)	1034 (55.1)	n/m	n/m	n/m		1131 (44.3)
Experienced physical IPV in past year	403 (59.5)	290 (15.4)	629 (37.4)	633 (45.3)	216 (23.2)		2171 (33.2)
Experienced non-partner sexual violence	228 (33.7)	54 (2.9)	n/m	n/m	n/m		282 (11.0)
**Men**	(n = 674)	(n = 1973)	(n = 1651)	(n = 1400)		(n = 2406)	(n = 8104)
Age group							
*18-25 years*	473 (70.2)	378 (19.2)	123 (7.5)	102 (7.3)		997 (41.4)	2073 (25.6)
*26-35 years*	199 (29.5)	560 (28.4)	760 (46.0)	695 (49.6)		1134 (47.1)	3348 (41.3)
*36-45 years*	2 (0.3)	456 (23.1)	589 (35.7)	480 (34.3)		275 (11.4)	1802 (22.2)
*> = 46 years*	0 (0.0)	579 (29.3)	179 (10.8)	123 (8.8)		0 (0.0)	881 (10.9)
Current marital status							
*Married*	22 (3.3)	1271 (64.4)	1095 (66.3)	885 (63.2)		448 (18.8)	3721 (46.1)
*In a relationship*	508 (75.4)	533 (27.0)	556 (33.7)*	515 (36.8)*		1539 (64.7)	3651 (45.2)
*No relationship*	144 (21.4)	169 (8.6)				391 (16.4)	704 (8.7)
Education							
*None*	0 (0.0)	372 (18.9)	265 (16.1)	241 (17.2)		0 (0.0)	878 (10.9)
*Primary school*	77 (11.4)	334 (16.9)	1088 (65.9)	913 (65.2)		140 (5.9)	2552 (31.6)
*Secondary school or above*	597 (88.6)	1267 (64.2)	298 (18)	246 (17.6)		2249 (94.1)	4657 (57.6)
Food insecurity							
*None/little*	138 (20.5)	1621 (82.2)	370 (22.4)	223 (15.9)		1213 (50.5)	3565 (44.0)
*Moderate*	179 (26.6)	232 (11.8)	731 (44.3)	600 (42.9)		536 (22.3)	2278 (28.1)
*Severe*	356 (52.9)	120 (6.1)	549 (33.3)	577 (41.2)		651 (27.1)	2253 (27.8)
Employed in the past three months	240 (35.7)	1231 (71.6)	n/m	n/m		1192 (50.0)	2663 (55.7)
Employed in the past year	156 (23.2)	1251 (63.4)	n/m	n/m		806 (33.7)	2213 (43.9)
Perpetrated physical IPV in past year	337 (50.2)	235 (11.9)	402 (24.4)	366 (26.3)		952 (39.7)	2292 (28.4)
Perpetrated non-partner sexual violence in past year	261 (38.8)	196 (9.9)	n/m	n/m		834 (34.8)	1291 (25.6)

RRS – Rural Response System, WFWI – Women for Women International, IPV – intimate partner violence, n/m – not measured

*Living together as married.

Across all studies with male samples, two-thirds of men (66.9%) were aged 18-35 years old. 46.1% of men were married to women and 45.2% were in a relationship but not married. Overall, only 8.7% of men across all studies were not in a relationship. Just over half of the men had secondary school education or above (57.6%) and just over half (55.7%) of the men in three studies were employed in the past three months (we did not ask about employment status in Rwanda) (**Table 3**). Among the 8104 men across the five studies, over a quarter of men (28.4%) reported perpetrating past-year intimate partner violence, with study-specific prevalence ranging from 11.9% (Ghana) to 50.2% (SSCF). Across all studies, 25.6% of men reported perpetrating past year NPSV, ranging from 9.9% in Ghana to 38.8% in SSCF. We did not ask about the perpetration of NPSV in Rwanda. Overall, 28.1% of men reported moderate food insecurity (range from 11.8% to 44.3%) and 27.8% of men reported severe food insecurity (range from 6.1% to 52.9%) (**Table 3**).

### Association between violence experience and food insecurity

**Table 4** shows the bi-variable associations between food insecurity and women’s experience of physical IPV. In all studies, there was a greater proportion of women reporting IPV across the categories of increasing food insecurity, compared to those reporting little or no food insecurity. Across all of the studies, women experiencing severe food insecurity, compared to those experiencing little or none, had more than a 2-fold increased risk of experiencing IPV in the past 12 months with adjusted associations ranging from an adjusted incidence rate ratio (aIRR) = 2.15 (95% CI = 1.56 to 2.96) in Ghana to aIRR = 2.39 (95% CI = 1.76 to 3.25) in the WFWI evaluation in Afghanistan. The risk associated with moderate food insecurity, compared with little or no food insecurity, was elevated in all studies except the RRS study in Ghana (aIRR = 1.29, 95% CI = 0.91 to 1.82). In the pooled analysis, women who had moderate insecurity, compared to those who had little or no food insecurity, had a 40% increase in risk of experiencing IPV in the past 12 months (aIRR = 1.40, 95% CI = 1.23 to 1.60) and those with severe food insecurity had a 73% increase in risk of experiencing IPV in the past 12 months, compared to those with little or no food insecurity (aIRR = 1.73, 95% CI = 1.41 to 2.12).

**Table 4:** Relationship between food insecurity, physical intimate partner violence (IPV) and non-partner sexual violence (NPSV) experience among women

		Experienced physical IPV in the past year					Experienced NPSV in the past year			
		**All**	**No**	**Yes**			**No**	**Yes**		
**Study**	**Food insecurity**	**n**	n (%)*	n (%)*	***P*-value**	**aIRR (95% CI)†**	**n (%)**	**n (%)**	***P*-value**	**aIRR (95% CI)†**
SSCF	None/little	144	83 (57.6)	61 (42.4)	<0.001	Ref.	109 (75.7)	35 (24.3)	0.027	Ref.
	Moderate	163	63 (38.7)	100 (61.4)		1.39 (1.01 to 1.91)	105 (64.4)	58 (35.6)		1.37 (0.89 to 2.09)
	Severe	370	128 (34.6)	242 (65.4)		1.45 (1.09 to 1.93)	235 (63.5)	135 (36.5)		1.25 (0.86 to 1.83)
Ghana	None/little	1536	1335 (86.9)	201 (13.1)	<0.001	Ref.	1496 (97.4)	40 (2.6)	0.304	Ref.
	Moderate	208	170 (81.7)	38 (18.3)		1.29 (0.91 to 1.82)	200 (96.1)	8 (3.9)		1.32 (0.62 to 2.83)
	Severe	133	82 (61.7)	51 (38.4)		2.15 (1.56 to 2.96)	127 (95.5)	6 (4.5)		1.18 (0.48 to 2.88)
Rwanda Couples	None/little	327	230 (70.3)	97 (29.7)	0.002	Ref.				
	Moderate	702	430 (61.3)	272 (38.8)		1.31 (1.04 to 1.65)				
	Severe	631	371 (58.8)	260 (41.2)		1.39 (1.10 to 1.76)				
Rwanda community	None/little	259	176 (68.0)	83 (32.1)	<0.001	Ref.				
	Moderate	621	340 (54.8)	281 (45.3)		1.42 (1.11 to 1.81)				
	Severe	519	250 (48.2)	269 (51.8)		1.62 (1.26 to 2.07)				
WFWI	None/little	567	484 (85.4)	83 (14.6)	<0.001	Ref.				
	Moderate	134	90 (67.2)	44 (32.8)		1.86 (1.27 to 2.73)				
	Severe	231	142 (61.5)	89 (38.5)		2.39 (1.76 to 3.25)				
Overall	None/little	2833	2308 (81.5)	525 (18.5)	<0.001	Ref.	1605 (95.5)	75 (4.5)	<0.001	Ref.
	Moderate	1828	1093 (59.8)	735 (40.2)		1.40 (1.23 to 1.60)	305 (82.2)	66 (17.8)		1.36 (0.94 to 1.96)
	Severe	1884	973 (51.6)	911 (48.4)		1.73 (1.41 to 2.12)	362 (72.0)	141 (28.0)		1.24 (0.88 to 1.76)

SSCF – Stepping Stones and Creating Futures intervention, IPV – intimate partner violence, NPSV – non-partner sexual violence, aIRR – adjusted incidence rate ratio, CI – confidence interval, WFWI – Women for Women International

*n (%): row percentage.

†aIRR (95% CI): adjusted for participant's age and childhood trauma experience.

Data on women’s experiences of non-partner sexual violence and food insecurity were available for two studies. In one of the two, the SSCF evaluation, there was an elevated likelihood of reporting NPSV associated with moderate or severe food insecurity on a bi-variable analysis (*P* = 0.027). The aIRR, however, was not elevated for either of the two studies, and notably the 95% confidence intervals were wide. The overall pooled effect showed a similar pattern.

There was a bi variable association between food insecurity and physical IPV perpetration for men in four of the five data sets for one of the levels of the food insecurity variable (**Table 5**). However, the risk, compared to participants with little or no food insecurity, was only clearly elevated for one level of the food insecurity variable in the Indashyikirwa couples’ study (severe insecurity, 38% increased risk), the Indashyikirwa community study (severe insecurity, 44% increased risk) and the Sonke Change trial (moderate insecurity, 27% increased risk). The dose-response relationship seen in the women’s data was not visible across the studies for men’s perpetration. Among men, the overall effect showed an increased risk of past year IPV perpetration associated with food insecurity. For moderate food insecurity, the risk was 42% greater, compared to those with little or no food insecurity (aIRR = 1.42, 95% CI = 1.23 to 1.64), and for severe food insecurity, the risk was 28% higher (aIRR = 1.28, 95% CI = 1.06 to 1.55). Across the individual studies, the pattern of associations was much more mixed.

**Table 5:** Relationship between food insecurity and physical intimate partner violence (IPV) and non-partner sexual violence (NPSV) perpetration among men

			Physical IPV perpetration in the past year				NPSV perpetration in the past year			
			**No**	**Yes**			**No**	**Yes**		
**Study**	**Food insecurity level**	**n**	n (%)*	n (%)*	***P*-value**	**aIRR (95% CI)†**	**n (%)**	**n (%)**	***P*-value**	**aIRR (95% CI)†**
SSCF	None/little	137	76 (55.5)	61 (44.5)	0.233	Ref.	85 (62.0)	52 (38.0)	0.831	Ref.
	Moderate	179	82 (45.8)	97 (54.2)		1.13 (0.82 to 1.56)	106 (59.2)	73 (40.8)		0.97 (0.67 to 1.40)
	Severe	355	176 (49.6)	179 (50.4)		0.97 (0.72 to 1.31)	219 (61.7)	136 (38.3)		0.82 (0.59 to 1.14)
Ghana	None/little	1621	1441 (88.9)	180 (11.1)	0.05	Ref.	1466 (90.4)	155 (9.6)	0.495	Ref.
	Moderate	232	194 (83.6)	38 (16.4)		1.14 (0.79 to 1.63)	205 (88.4)	27 (11.6)		0.98 (0.64 to 1.49)
	Severe	120	103 (85.8)	17 (14.2)		1.34 (0.81 to 2.2)	106 (88.3)	14 (11.7)		1.33 (0.77 to 2.31)
Rwanda Couples	None/little	369	298 (80.8)	71 (19.2)	0.025	Ref.				
	Moderate	729	545 (74.8)	184 (25.2)		1.30 (0.99 to 1.71)				
	Severe	547	400 (73.1)	147 (26.9)		1.38 (1.04 to 1.83)				
Rwanda community	None/little	222	178 (80.2)	44 (19.8)	0.027	Ref.				
	Moderate	595	440 (74.0)	155 (26.1)		1.30 (0.93 to 1.81)				
	Severe	573	406 (70.9)	167 (29.1)		1.44 (1.03 to 2.01)				
Sonke	None/little	1208	805 (66.6)	403 (33.4)	<0.001	Ref.	842 (69.6)	367 (30.4)	<0.001	Ref.
	Moderate	536	272 (50.8)	264 (49.3)		1.27 (1.08 to 1.49)	308 (57.5)	228 (42.5)		1.14 (0.96 to 1.35)
	Severe	648	365 (56.3)	283 (43.7)		1.09 (0.93 to 1.28)	407 (63.1)	238 (36.9)		0.96 (0.80 to 1.14)
Overall	None/little	3557	2798 (78.7)	759 (21.3)		Ref.	2393 (80.7)	574 (19.3)	<0.001	Ref.
	Moderate	2271	1533 (67.5)	738 (32.5)		1.24 (1.11 to 1.39)	619 (65.4)	328 (34.6)		1.09 (0.94 to 1.26)
	Severe	2243	1450 (64.6)	793 (35.4)		1.18 (1.02 to 1.37)	732 (65.4)	388 (34.6)		0.95 (0.82 to 1.10)

SSCF – Stepping Stones and Creating Futures intervention, IPV – intimate partner violence, aIRR – adjusted incidence rate ratio, CI – confidence interval

*n (%): row percentage.

†aIRR (95% CI): adjusted for participant's age and childhood trauma experience.

Data on men’s reports of perpetration of non-partner sexual violence and food insecurity were available for three studies and a statistically significant association was only seen for the Sonke Change Trial and the moderate food insecurity exposure. The overall effect was significant for the bi variable association but risk elevation was not shown in the adjusted analysis (the aIRR).

**Table 6** presents associations between IPV and NPSV experience and perpetration and food insecurity with food insecurity treated as a two-level variable (none vs moderate or severe food insecurity). The overall effect for women for IPV experience associated with food insecurity showed a 58% increase in risk (aIRR = 1.58, 95% CI = 1.35 to 1.85). For NPSV experienced by women, the overall effect suggested an elevation in risk (aIRR = 1.27, 95% CI = 0.93 to 1.74), but 95% confidence intervals overlapped 1.00. The individual studies show the same pattern.

**Table 6:** Effect of moderate/severe food insecurity on risk of physical intimate partner violence (IPV) and non-partner sexual violence (NPSV) experience/perpetration

	Women's risk of experiencing violence		Men's risk of violence perpetration	
	**Physical IPV**	**Non-partner sexual**	**Physical IPV**	**Non-partner sexual**
**Study**	aIRR (95% CI)*	aIRR (95% CI)*	aIRR (95% CI)*	aIRR (95% CI)*
South Africa SSCF	1.43 (1.09 to 1.88)	1.28 (0.89 to 1.84)	1.02 (0.77 to 1.36)	0.86 (0.63 to 1.18)
Ghana RRS	1.65 (1.28 to 2.13)	1.24 (0.67 to 2.31)	1.20 (0.88 to 1.63)	1.07 (0.75 to 1.52)
Rwanda Indashyikirwa couples	1.35 (1.09 to 1.67)	n/m	1.33 (1.03 to 1.72)	n/m
Rwanda Indashyikirwa community	1.51 (1.20 to 1.90)	n/m	1.37 (1.00 to 1.87)	n/m
Afghanistan WFWI	2.20 (1.65 to 2.93)	n/m	n/m	n/m
South Africa Sonke Change	n/m	n/m	1.17 (1.02 to 1.34)	1.05 (0.91 to 1.12)
Overall effect	1.58 (1.35 to 1.85)	1.27 (0.93 to 1.74)	1.19 (1.08 to 1.32)	1.02 (0.90 to 1.15)

SSCF – Stepping Stones and Creating Futures intervention, IPV – intimate partner violence, aIRR – adjusted incidence rate ratio, CI – confidence interval, RRS – Rural Response System, WFWI – Women for Women International, n/m – not measured

*aIRR: Models adjusted for participant's age and childhood trauma experience.

The overall effect for men shows a 19% increased risk of physical IPV perpetration associated with food insecurity (aIRR = 1.19, 95% CI = 1.08 to 1.32). The same pattern was seen in four of the five data sets. There does not appear to be an association between NPSV perpetration by men and food security, aIRR = 1.02 (95% CI = 0.90 to 1.15). This pattern was reflected in the three studies with data on NPSV perpetration.

## DISCUSSION

Our analysis has shown a close and consistent association between food insecurity and IPV experience, with a clear dose-response relationship shown between the degree of food insecurity and the likelihood of women experiencing IPV. Additionally, we have demonstrated a generally, positive relationship, between food insecurity and men's reported IPV perpetration, but we did not demonstrate a dose-response relationship, and the pattern was inconsistent between studies. We found evidence to suggest that women who were food insecure may have had an elevated risk of experiencing NPSV, but the findings did not achieve statistical significance, partly due to the small number of events. There was no evidence that food insecurity experienced by men increased their risk of perpetrating non-partner sexual violence. To our knowledge, this paper presents the first published meta-analysis of food insecurity and IPV and NPSV using comparable measures and with primary data analysis.

The conclusion that there was an elevated risk of IPV associated with food insecurity has been supported by studies in other settings [7,31-35]. It was also the conclusion of the meta-analysis of Hatcher et al. [6], who reported associations in 18 studies between women’s experiences of assorted IPV and food insecurity measures, concluding that a binary measure of food insecurity was significantly associated with the main study group of women or a sub-group, in all. They also concluded that there were similar findings for men’s perpetration of IPV and food insecurity, although in two of the four studies, and among sub-groups in another, there was no significant association. Some of the studies reported in Hatcher et al. [6] are also reported in our paper, but there are differences in the effect sizes due to our conducting primary data analysis and categorising the variables differently.

There are several hypothesised pathways through which poverty increases women’s vulnerability to being subject to NPSV. The first through the impact of poverty and living in impoverished neighbourhoods on housing quality, neighbourhood risks and safety, and access to safe transport, all of which impact NPSV vulnerability [36]. The second through the elevated risks of women living in poverty having other trauma exposure, including previous rape and other sexual abuse, and risky sexual partnerships which may impact on their alcohol consumption and drug use, and transactional sex or sex work engagement, all of which may increase the risk of NPSV by influencing how women interpret cues and danger signs [37]. A few other studies that have examined the association between food insecurity and NPSV experience and perpetration. One study from the USA found significant positive associations between NPSV experience and food insecurity reported by both women and men, with the associations stronger for women [7]. However, two studies from South Africa have found no association between food insecurity and women’s experience of rape and men’s perpetration of NPSV [38,39]. It was notable that the study with women only had a single variable to measure food insecurity, which may have been an insufficiently sensitive measure. The study with men, however, showed that sexual entitlement was a more important driver of NPSV perpetration than poverty, and this was confirmed in a third study that found that men who were more food secure and economically advantaged were more likely to perpetrate NPSV [40]. Taken together, these studies support our conclusion that there is no consistent link between food insecurity and NPSV perpetration, as well as insufficient evidence on the relationship between NPSV experience and food insecurity.

The myriad of ways in which poverty, as indicated by food insecurity, impact relationships and IPV risk are described above. There are many measures of poverty. However, in LMICs food insecurity is very straight forward indicator to measure in research and conceptually much clearer than measures of assets and income [20]. It is a strength of this analysis that almost the same measure of food insecurity was used in all settings analysed, and that comparable measures of NPSV and IPV were used. This addresses the limitation of previous studies on associations between food insecurity and IPV and NPSV, which have tended to focus on IPV only, or to use diverse measures or coding for both food insecurity and violence outcomes. The study also contributes to the sparse body of knowledge in low- to middle-income settings, where extreme poverty and VAWG commonly co-occur.

We also acknowledge some limitations in the research. The cross-sectional nature of the current data limits our ability to draw any conclusions about the temporal relationships. There is to date just one contribution to the literature from a longitudinal analysis, which supports the general findings in respect of male perpetration of IPV and food insecurity [31]. Only one of the five studies (Ghana) were population-based and other studies were limited in their generalizability, as they were based on populations recruited for the impact evaluation. There may also have been issues with measurement of key variables related to social desirability of violence experience or exposure and food insecurity, although these would likely have attenuated the association, and we used the “gold standard” measures of violence experience and perpetration.

## CONCLUSIONS

Our analysis shows a strong association between food insecurity and the experience and perpetration of physical intimate partner violence reported by men and women, with the overall associated risk among women being elevated by 58%, and a greater risk for women with more severe food insecurity. This clearly shows the importance of understanding the potential to reap benefits in violence reduction from poverty reduction programmes. We did not show an increased risk of NPSV experience associated with food insecurity although the analysis suggested that more food-insecure women may have been more vulnerable to NPSV. Further our findings did not show that food insecure men were more likely to perpetrate NPSV than other men. These observations underscore the importance of viewing food insecurity as a risk factor for IPV and addressing it in IPV prevention interventions in affected populations. Thus far there have been very positive findings from a range of studies examining the impact on IPV of cash transfers, and combined interventions with microfinance, or other livelihood strengthening approaches, and gender transformative interventions [41]. This body of work has shown reductions in IPV associated with these interventions, but the extent to which these are sustained and benefit impacts children in the households still requires further research. As does the intervention dose and time required to reduce the IPV experience of all beneficiaries. Our research has also highlighted a difference in risk factors for IPV and NPSV and remind VAWG researchers and programmers that it is important to understand the drivers and risk factors for NPSV as being somewhat different from those for IPV, even if there are significant areas of overlap.
